# Pediatric Dermatoses in Western India: A Clinicoetiological Study From a Tertiary Healthcare Center in the Saurashtra Region of Gujarat

**DOI:** 10.7759/cureus.79656

**Published:** 2025-02-25

**Authors:** Raghavon U N, Ashma Surani, Neela Bhuptani, Bharti Patel

**Affiliations:** 1 Dermatology, St. Peter's Medical College, Hosur, IND; 2 Dermatology, Byramjee Jeejeebhoy (BJ) Medical College, Ahmedabad, IND; 3 Dermatology, Pandit Deendayal Upadhyay Medical College, Rajkot, IND

**Keywords:** children, dermatology, eczema, papulosquamous disorders, pediatric dermatoses, skin infections

## Abstract

Background

Pediatric dermatoses are among the most common dermatologic conditions seen in clinical practice. These conditions often present with distinct characteristics compared to adult skin disorders, and their prevalence and clinical patterns can vary based on factors such as environmental conditions, socioeconomic status, genetics, and cultural influences.

Objective

This study aimed to assess the prevalence and clinical manifestations of pediatric dermatoses in children aged 0-14 years in the Saurashtra region of Gujarat.

Materials and methods

This hospital-based descriptive study was conducted at a tertiary care center in Rajkot, Gujarat, over a 20-month period. A total of 500 pediatric patients (264 boys (52.8%) and 236 girls (47.2%)) were included. The patients were categorized into four age groups: neonates (up to 28 days) (47 cases (9.4%)), infants (28 days to one year) (81 cases (16.2%)), preschool children (1-6 years) (180 cases (36%)), and school-going children (6-14 years) (192 cases (38.4%)). A detailed clinical examination was performed, and necessary diagnostic procedures were conducted as needed.

Results

The male-to-female ratio was 1.12:1. Infections and infestations were the most prevalent dermatoses, observed in 185 cases (37%). Bacterial infections were the most common subtype (60 cases (32.4% of infections)), followed by fungal infections (52 cases (28.1% of infections)) and viral infections (45 cases (24.3% of infections)). Eczematous disorders were the second most common group, accounting for 67 cases (13.4%), with pityriasis alba being the leading type (21 cases (31.3% of eczema)). Papulosquamous disorders constituted 48 cases (9.6%). Other categories included disorders of keratinization (31 cases (6.2%)) and vitiligo (32 cases (6.4%)).

Conclusion

This study highlights the variability in dermatological prevalence across different pediatric age groups. The findings illustrate the importance of early recognition and diagnosis of the most common pediatric dermatoses, which will help improve management strategies among general practitioners, pediatricians, and dermatologists.

## Introduction

Children's skin is much different from adult skin, thus causing some different dermatological conditions among the pediatric population. The anatomical and physiological properties of children's skin give rise to these varied clinical manifestations seen in pediatric dermatoses [1]. The distribution and prevalence of these diseases vary due to geographical, socioeconomic, climatic, hygiene-related, and genetic predisposition factors [2,3]. Pediatric skin diseases form a major percentage of both pediatric and dermatological outpatient consultations, even up to 30% of all consultations [4].

Factors such as age, gender, and environment influence the epidemiology of pediatric skin diseases, therefore causing different disease patterns within different regions [5,6].

Environmental factors such as climate and hygiene, along with genetic predispositions, play a significant role in the etiology of pediatric skin disorders [7,8]. This highlights the need for specific research and customized public health interventions, which should center on the early diagnosis and proper management of skin disorders in children. This research intends to present a statistical view of common dermatological diseases in children within the Saurashtra region of Gujarat.

## Materials and methods

This hospital-based, descriptive study was conducted over a 20-month period from February 2019 to October 2020 in the Dermatology Department of Pandit Deendayal Upadhyay Civil Hospital located in Rajkot, Gujarat, India. The study was designed to assess the prevalence and clinical patterns of pediatric dermatoses in a representative cohort from urban, semi-urban, and rural populations. Ethical approval for the study was obtained from the Institutional Ethics Committee of Pandit Deendayal Upadhyay (PDU) Medical College (approval number: PDU/MCR/IEC/1262/2019) on January 24, 2019.

We included consecutive patients attending our outpatient department (OPD) aged 0-14 years of either gender, presenting with at least one skin manifestation, by purposive sampling. Informed written consent was obtained from parents or guardians before enrolling their children in the study.

Children aged 0-14 years with dermatological conditions presenting at the dermatology OPD were included in the study. In contrast, children above the age of 14 years were excluded from the study. Participants were categorized into four age groups: neonates (up to 28 days), infants (28 days to one year), preschool children (1-6 years), and school-going children (6-14 years). 

Data collection involved a pre-designed pro forma to document demographic details, history of the presenting condition, past medical and family history, and treatment history. The onset, duration, progression, and associated symptoms of skin lesions were meticulously recorded. Clinical examination was performed for all participants, with findings documented systematically.

Clinical examinations were performed for all patients, and diagnostic investigations were conducted as needed. Diagnostic tests including potassium hydroxide (KOH) mount, Gram staining, culture and sensitivity tests, Wood's lamp examination, skin biopsy, and relevant hematological or biochemical evaluations were also performed when indicated. Dermatoses were classified according to the Tenth Revision of the International Statistical Classification of Diseases (ICD-10). The recorded skin conditions were systematically categorized into 15 etiological groups: physiological conditions, infections and infestations, nevi and related disorders, papulosquamous disorders, disorders of keratinization, eczematous disorders, genodermatoses, immunobullous diseases, nutritional and metabolic disorders, urticaria, vitiligo, drug eruptions, alopecia, connective tissue disorders, and miscellaneous conditions.

Data were analyzed using descriptive statistics with IBM SPSS Statistics for Windows, Version 23.0 (Released 2015; IBM Corp., Armonk, New York, United States).

## Results

Age and sex distribution

A total of 500 pediatric patients with dermatological conditions were included in the study. The male-to-female ratio was 1.12:1, with boys accounting for 52.8% (n=264) and girls 47.2% (n=236). The highest number of cases was observed in the school-age group (6-14 years), constituting 38.4% of cases, followed by preschool children (1-6 years) at 36%, infants (28 days-1 year) at 16.2%, and neonates (up to 28 days) with the lowest prevalence at 9.4%. Table 1 illustrates the distribution of cases by age and sex.

**Table 1:** Distribution of cases according to age and sex

Group	Age	Boys	Girls	Total	Percentage (%)
Neonates	Up to 28 days	26	21	47	9.4
Infants	28 days-1 year	40	41	81	16.2
Preschool children	1-6 years	85	95	180	36
School-going children	6-14 years	113	79	192	38.4
Total		264	236	500	100
Percentage		52.8	47.2		

The etiological classification revealed infections and infestations (Table 2) as the most common group, accounting for 37% of cases (n=185). Eczematous disorders followed with 13.4% (n=67), while papulosquamous disorders represented 9.6% (n=48). Less common etiologies included physiological lesions (6.6%; n=33), disorders of keratinization (6.2%; n=31), and nutritional/metabolic conditions (1.6%; n=8).

**Table 2:** Classification according to the etiology of diseases

Sr. no.	Disorders	No. of cases	Percentage (%)
1	Physiological conditions	33	6.6
2	Infections and infestations	185	37
3	Nevi and related disorders	26	5
4	Papulosquamous disorders	48	9.6
5	Keratinization disorders	31	6.2
6	Eczematous disorders	67	13.4
7	Genodermatoses	10	2
8	Immunobullous diseases	2	0.4
9	Nutritional and metabolic disorders	8	1.6
10	Urticaria	28	5.6
11	Vitiligo	32	6.4
12	Drug eruptions	8	1.6
13	Alopecia	15	3
14	Connective tissue disorders	2	0.4
15	Miscellaneous conditions	5	1

Infectious dermatoses

**Figure 1:**
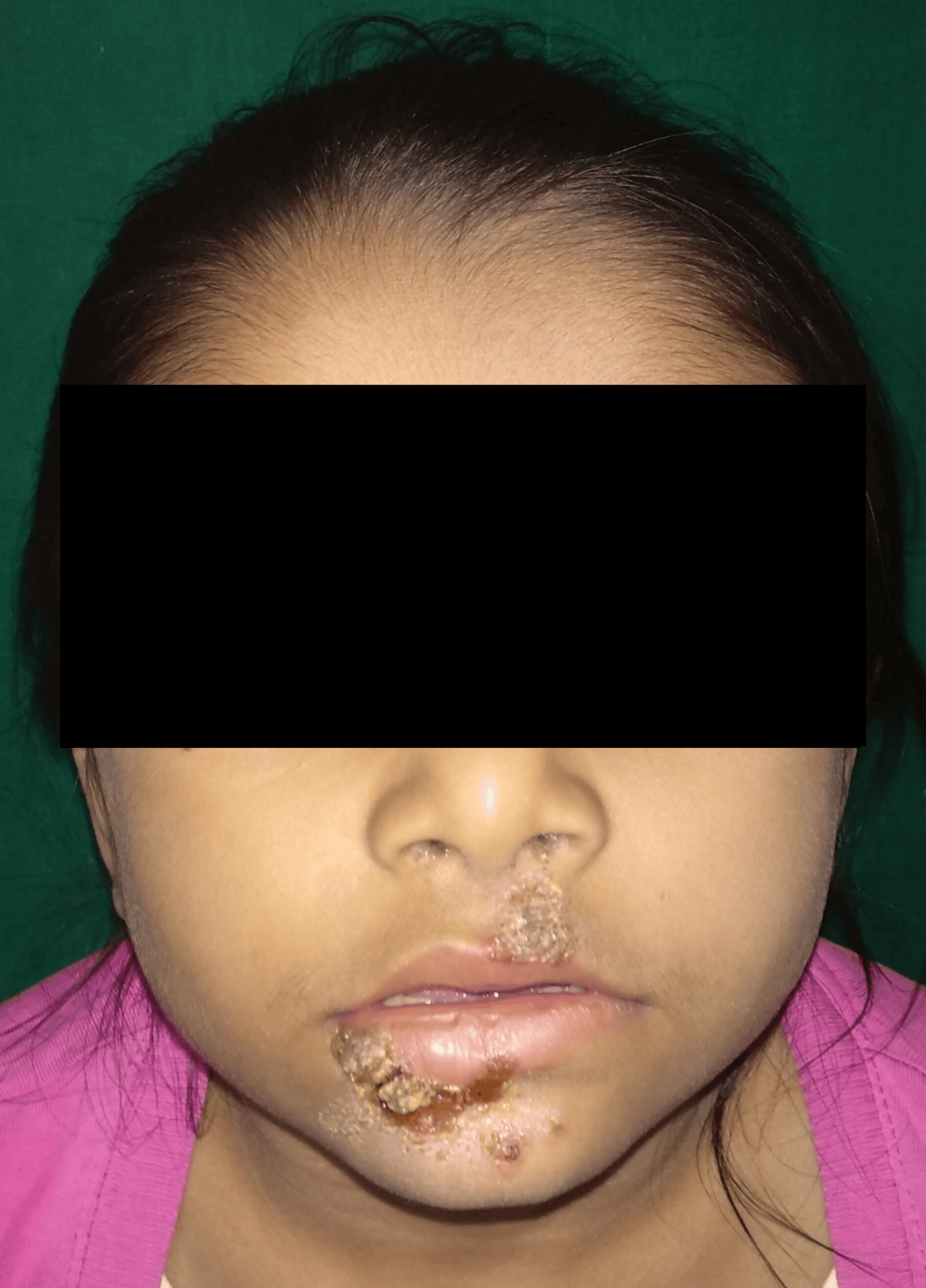
Impetigo: honey-colored crusted plaque seen

**Figure 2:**
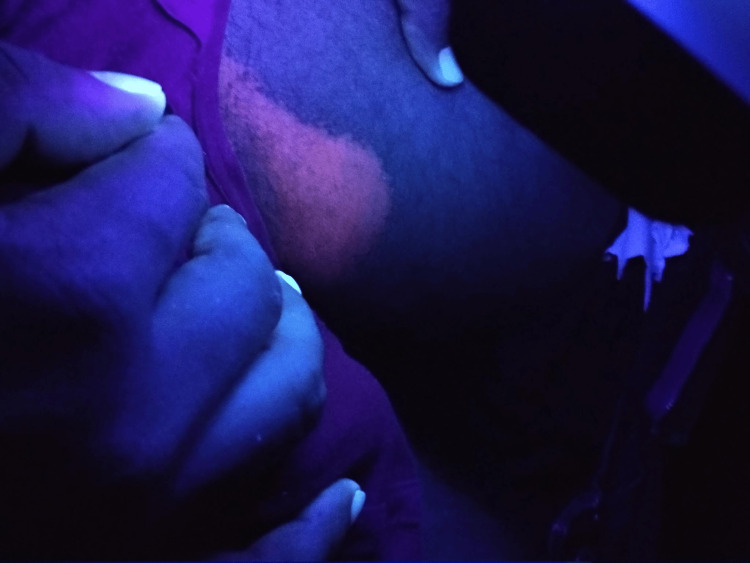
Erythrasma diagnosed utilizing Wood's lamp examination

**Figure 3:**
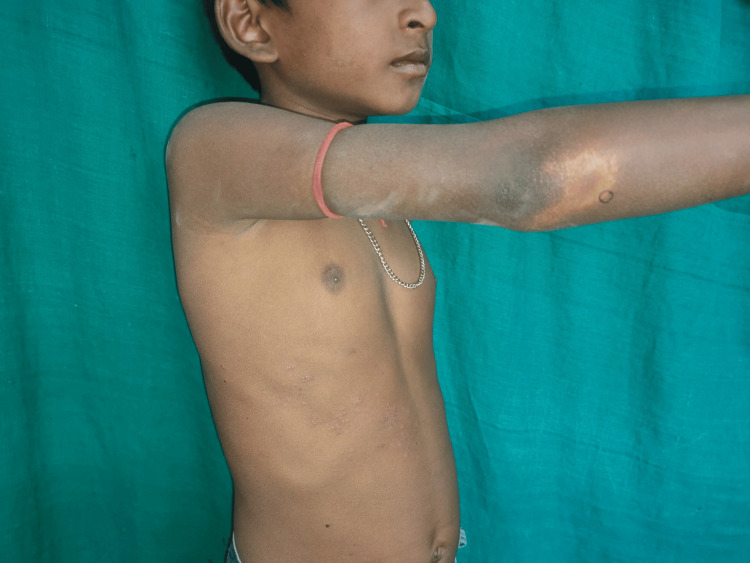
A case of tuberculoid leprosy presenting with hypopigmented patch

Among infectious dermatoses, bacterial infections were predominant (32.4%; n=60). Impetigo was the leading bacterial condition, constituting 48.3% of cases, followed by folliculitis (5%) and furuncle (3.4%). Fungal infections comprised 28.1% (n=52) of cases, with tinea corporis/cruris being the most common (50%), followed by candidiasis (17.3%) and pityriasis versicolor (13.4%). Viral infections accounted for 24.3% (n=45), with molluscum contagiosum (26.6%) as the most prevalent, followed by hand, foot, and mouth disease (24.4%) and viral warts (22.2%). Parasitic infestations were primarily scabies (92.8%; n=26). Table 3 depicts the proportions of infections and infestations recorded in our study. Figures 1-3 show the clinical images of specific infections (impetigo, erythrasma, and tuberculoid leprosy) diagnosed in the study.

**Table 3:** Distribution of cases according to the type of infection/infestation

Type of infection/infestation	No. of cases	Percentage (%)
Bacterial infection	60	32.4
Fungal infection	52	28.1
Viral infection	45	24.3
Parasitic infestation	28	15.15
Total	185	37 of total cases

Eczematous disorders

Eczematous disorders constituted 13.4% (n=67) of the total dermatological cases observed in the study. Among these, pityriasis alba was the most prevalent condition, accounting for 31.3% (n=21) of the cases. This condition primarily affected children in the school-age group, presenting as hypopigmented, scaly patches, predominantly on the face and upper limbs. Seborrheic dermatitis was the second most common eczematous condition, observed in 29.9% (n=20) of cases. It frequently involved the scalp, face, and chest, with peaks during colder months. Atopic dermatitis contributed to 28.4% (n=19) of eczematous cases. This condition showed marked seasonal variation, with exacerbations predominantly in the winter months, and was often associated with a personal or family history of atopy. Other less common eczematous disorders included diaper dermatitis (4.5%; n=3), pompholyx (4.5%; n=3), and perioral dermatitis (1.5%; n=1). Table 4 provides a detailed distribution of the various eczematous disorders recorded in the study.

**Table 4:** Distribution of cases according to the different types of eczematous disorders in the present study

Eczematous disorders	No. of cases	Percentage (%)
Pityriasis alba	21	31.3
Seborrheic dermatitis	20	29.9
Atopic dermatitis	19	28.4
Diaper dermatitis	3	4.5
Pompholyx	3	4.5
Perioral dermatitis	1	1.5
Total	67	13.4 of total cases

Disorders of keratinization

Disorders of keratinization were observed in 6.2% (n=31) of the total cases (Table 5). These disorders encompassed a diverse group of conditions, with follicular keratosis emerging as the most common subgroup, accounting for 38.7% (n=12) of the cases. Within this subgroup, phrynoderma was the most frequently diagnosed condition (19.35%; n=6), followed by lichen spinulosus (16.12%; n=5) and keratosis pilaris (3.22%; n=1).

**Table 5:** Distribution of cases according to the different types of keratinization disorders in the present study KID: keratitis-ichthyosis-deafness

Disorder	No. of cases	Percentage (%)
Mendelian disorders of cornification		
Ichthyosis vulgaris	4	12.9
X-linked ichthyosis	3	9.6
Lamellar ichthyosis	3	9.6
Total	10	32.25
Follicular keratosis		
Phrynoderma	6	19.35
Lichen spinulosus	5	16.12
Keratosis pilaris	1	3.22
Total	12	38.7
Palmoplantar keratoderma	7	22.58
Progressive symmetric erythrokeratoderma	1	3.22
KID syndrome	1	3.22
Total	31	6.2 of total cases

Mendelian disorders of cornification comprised 32.25% (n=10) of keratinization disorders, including ichthyosis vulgaris (12.9%; n=4), X-linked ichthyosis (9.6%; n=3), and lamellar ichthyosis (9.6%; n=3). These conditions were predominantly seen in school-aged children and were associated with significant cosmetic concerns.

The third major category was palmoplantar keratoderma, contributing to 29.03% (n=9) of keratinization disorders. This group included palmoplantar keratoderma itself (22.58%; n=7), progressive symmetric erythrokeratoderma (3.22%; n=1), and keratitis-ichthyosis-deafness (KID) syndrome (3.22%; n=1).

Papulosquamous disorders

Papulosquamous disorders accounted for 9.6% (n=48) of the total dermatological cases observed in the study. Among these, pityriasis rosea was the most prevalent, comprising 31.2% (n=15) of the cases. It primarily affected children in the school-age group and presented with characteristic oval, scaly plaques, predominantly on the trunk.

Psoriasis vulgaris was the second most common papulosquamous condition, accounting for 25% (n=12) of cases. It was observed across various age groups, with a slight male predominance, and commonly involved the scalp, elbows, and knees.

Lichen planus represented 22.9% (n=11) of the cases. The most frequently affected sites included the wrists, legs, and oral mucosa. The condition was more commonly reported in older children, with no significant gender preference.

Other papulosquamous conditions included lichen striatus (8.3%; n=4), lichen nitidus (6.25%; n=3), and pityriasis lichenoides chronica (6.25%; n=3). These conditions were relatively rare but presented with chronicity and required prolonged follow-up.

Table 6 provides a detailed breakdown of the various papulosquamous disorders documented in the study.

**Table 6:** Distribution of cases according to the different types of papulosquamous disorders in the present study

Disorder	No. of cases	Percentage (%)
Pityriasis rosea	15	31.2
Psoriasis vulgaris	12	25
Lichen planus	11	22.9
Lichen striatus	4	8.3
Lichen nitidus	3	6.25
Pityriasis lichenoides chronica	3	6.25
Total	48	9.6 of total cases

Nevi and related disorders

Nevi and nevoid conditions were observed in 5.2% (n=26) of the total cases, comprising a variety of epidermal, melanocytic, and vascular lesions. These conditions were further classified into epidermal nevi and vascular lesions, with their respective subtypes and prevalence detailed in Table 7.

**Table 7:** Distribution of cases according to the different types of nevi and nevoid conditions in the present study

Disorder	No. of cases	Percentage (%)
Epidermal nevi		
Linear verrucous epidermal nevus	3	11.5
Congenital melanocytic nevi	3	11.5
Nevus depigmentosus	3	11.5
Linear and whorled nevoid hypomelanosis	1	3.8
Nevus sebaceous	1	3.8
Hypomelanosis of Ito	1	3.8
Becker's nevi	1	3.8
Nevus of Ota	1	3.8
Syringocystadenoma papilliferum	1	3.8
Total	15	57.7
Vascular lesions		
Infantile hemangioma	9	34.6
Capillary hemangioma	2	7.6
Total	11	42.3

Epidermal Nevi

**Figure 4:**
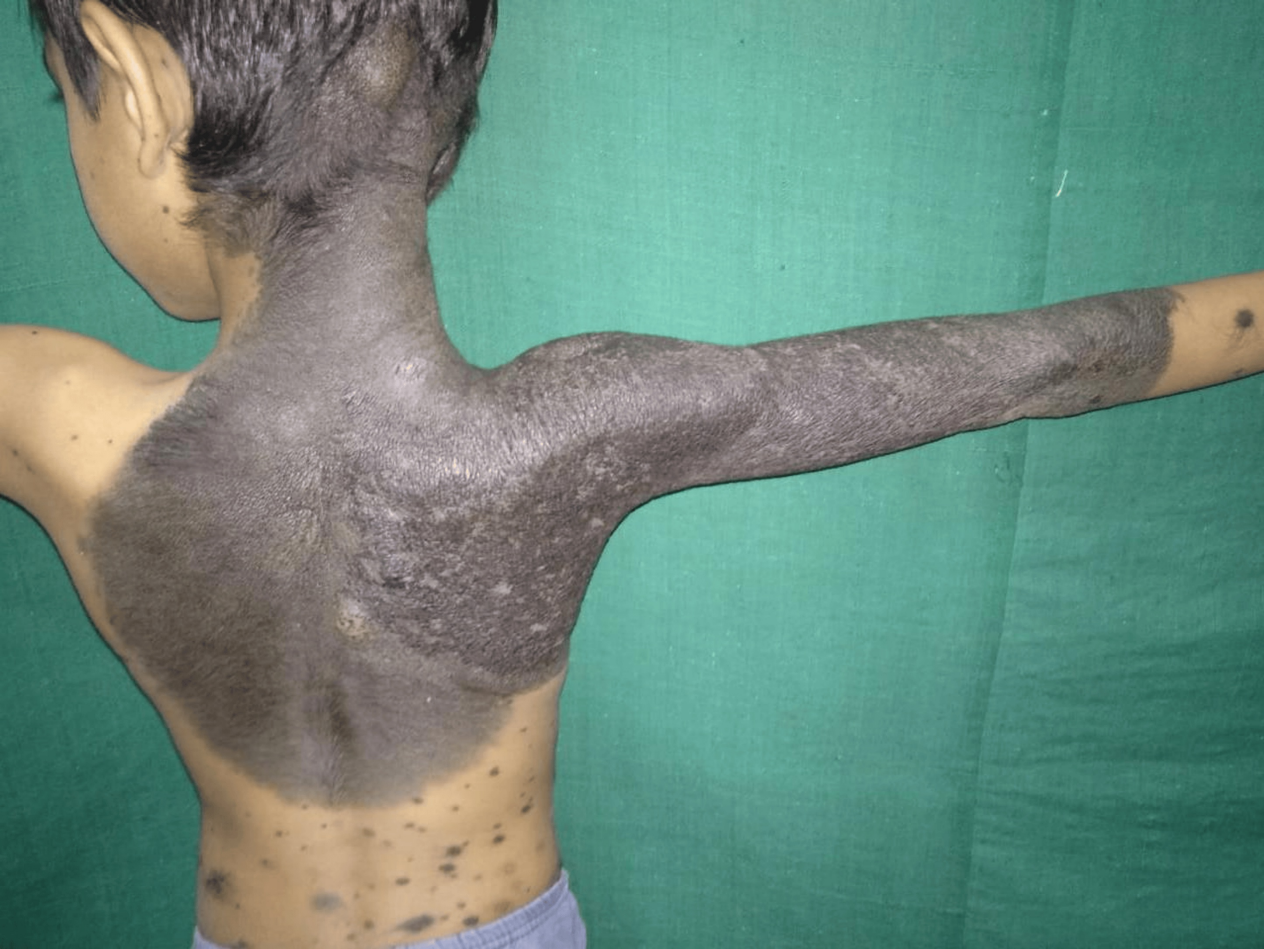
Giant congenital melanocytic nevus

**Figure 5:**
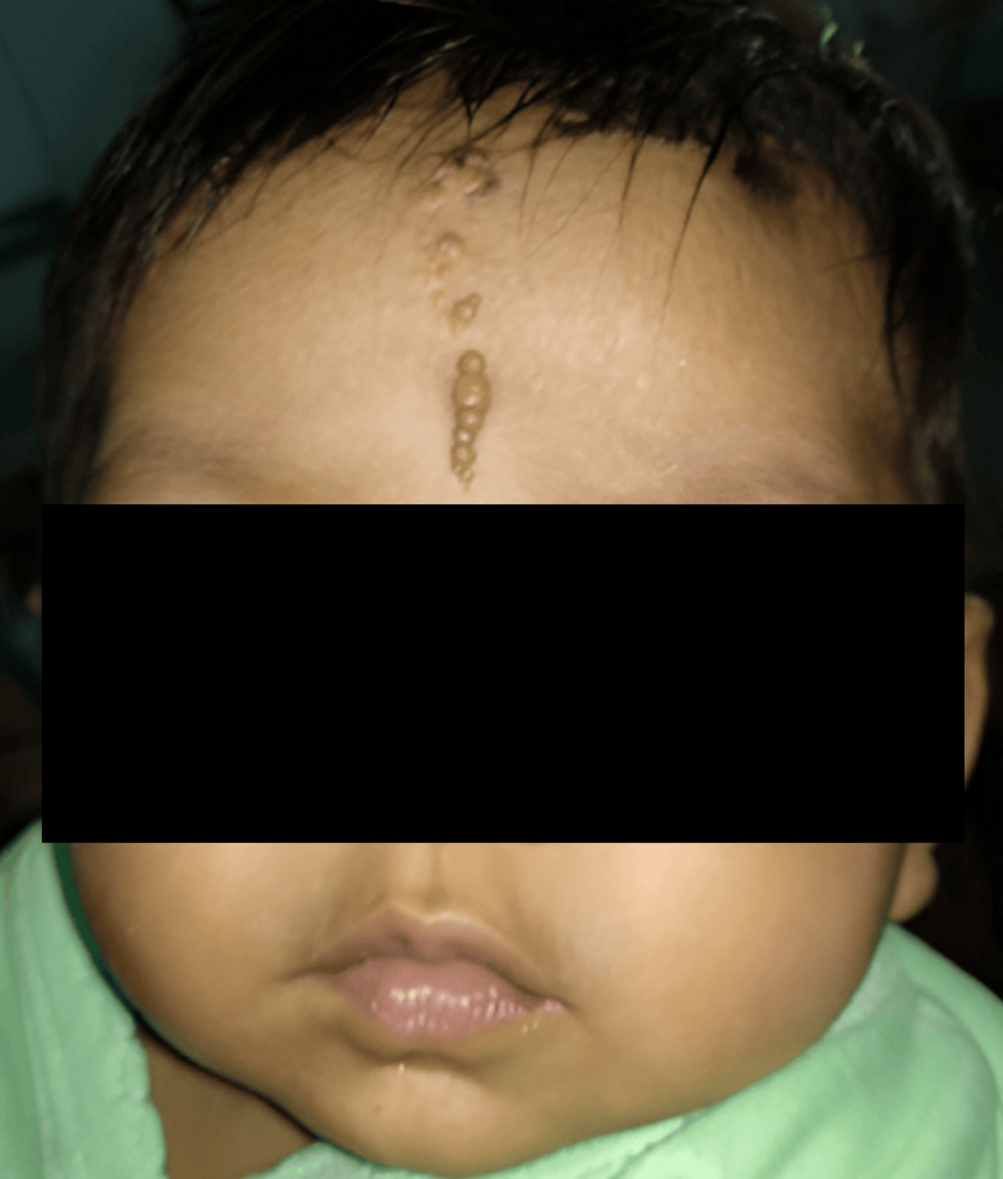
Syringocystadenoma papilliferum

Epidermal nevi accounted for 57.7% (n=15) of nevoid conditions. Among these, linear verrucous epidermal nevus, congenital melanocytic nevi (Figure 4), and nevus depigmentosus were the most frequent, each contributing 11.5% (n=3). Rare conditions such as linear and whorled nevoid hypomelanosis, nevus sebaceous, hypomelanosis of Ito, Becker's nevus, nevus of Ota, and syringocystadenoma papilliferum (Figure 5) were each seen in 3.8% (n=1) of cases.

Vascular Lesions

Vascular lesions represented 42.3% (n=11) of nevoid conditions. Among these, infantile hemangioma was the predominant subtype, accounting for 34.6% (n=9). Capillary hemangioma was less common, observed in 7.6% (n=2) of cases.

Autoimmune vesiculobullous diseases

Autoimmune vesiculobullous diseases were rare, with only two cases (0.4%) identified in the study. One case each of chronic bullous disease of childhood and childhood pemphigus vulgaris were observed.

Drug eruptions

Drug eruptions were identified in 1.6% (n=8) of the total cases, with various clinical presentations observed. The most common type was fixed drug eruption (FDE), accounting for 62.5% (n=5) of the cases. Other less frequent manifestations included toxic epidermal necrolysis (TEN), acute generalized eruptive pustulosis (AGEP), and maculopapular rash, each contributing 12.5% (n=1) to the total cases. This data highlights the need for vigilant identification of drug-related cutaneous adverse reactions in pediatric populations to prevent complications and improve outcomes.

Vitiligo

Vitiligo cases constituted 6.4% (n=32) of the total cases in the study, with three distinct clinical variants identified. The most common variant observed was vitiligo vulgaris, accounting for 78.1% (n=25) of the cases. This was followed by focal vitiligo, which contributed 18.8% (n=6). A single case (3.1%) of mucosal vitiligo was also reported.

Alopecia

Alopecia was observed in 3% (n=15) of the total cases in the study, with four distinct clinical types identified. The most prevalent type was alopecia areata, accounting for 60% (n=9) of cases, followed by tractional alopecia at 26.7% (n=4). Less commonly observed forms included trichotillomania and scarring alopecia due to pyoderma, each contributing 6.7% (n=1) to the total cases.

Urticaria

Urticaria accounted for 28 cases (5.6%), with acute urticaria being the predominant type (20 cases). Chronic urticaria was observed in one case.

Connective tissue diseases

Two cases (0.4%) of connective tissue diseases were documented, comprising one case each of mixed connective tissue disease and a mixed variant of morphea.

Nutritional and metabolic disorders

**Figure 6:**
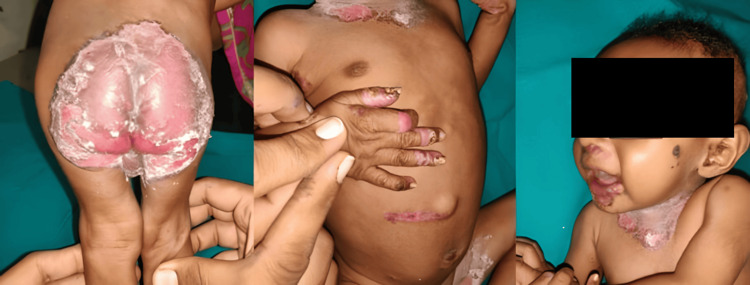
Acrodermatitis enteropathica

Nutritional and metabolic disorders constituted 1.6% of cases. Phrynoderma was the most common nutritional disorder (six cases), while metabolic disorders included one case each of acanthosis nigricans and acrodermatitis enteropathica (Figure 6).

Genodermatoses

Ten cases (2%) of genodermatoses were observed, with epidermolysis bullosa being the most common (five cases, 50%). Other conditions included tuberous sclerosis (two cases) and one case each of neurofibromatosis, incontinentia pigmenti, and pachyonychia congenita.

Physiological lesions

Physiological lesions accounted for 6.6% (n=33) of the total cases in the study. Various types of lesions were observed, with miliaria rubra being the most common, constituting 33.4% (n=11) of the cases, followed by miliaria pustulosa at 27.2% (n=9). Other notable conditions included miliaria crystallina (15.15%; n=5), Mongolian spots (9%; n=3), and neonatal acne (9%; n=3). Additionally, erythema toxicum neonatorum was seen in 6% (n=2) of the cases. The prevalence of physiological lesions, particularly in neonates, emphasizes their common and benign nature, often requiring minimal or no medical intervention.

Miscellaneous conditions

**Figure 7:**
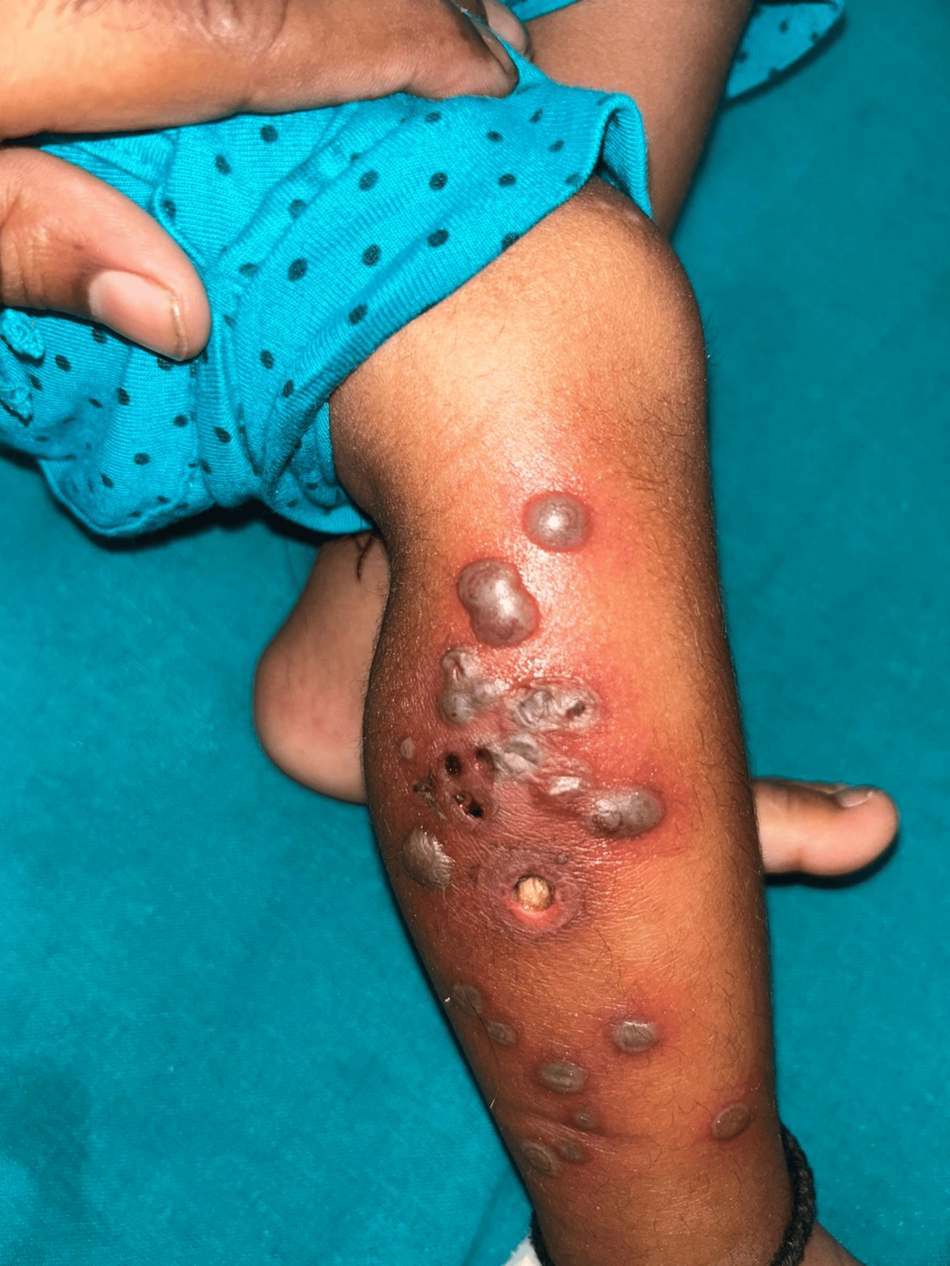
Bullous pyoderma gangrenosum child with underlying hematological malignancy

In this study, miscellaneous conditions included two cases of twenty-nail dystrophy, one case of leukemia cutis, one case of bullous pyoderma gangrenosum, and one case of piebaldism. It is important to remain vigilant when encountering bullous pyoderma gangrenosum, as it can be linked to acute Lymphocytic Leukemia, as shown in Figure 7.

**Figure 8:**
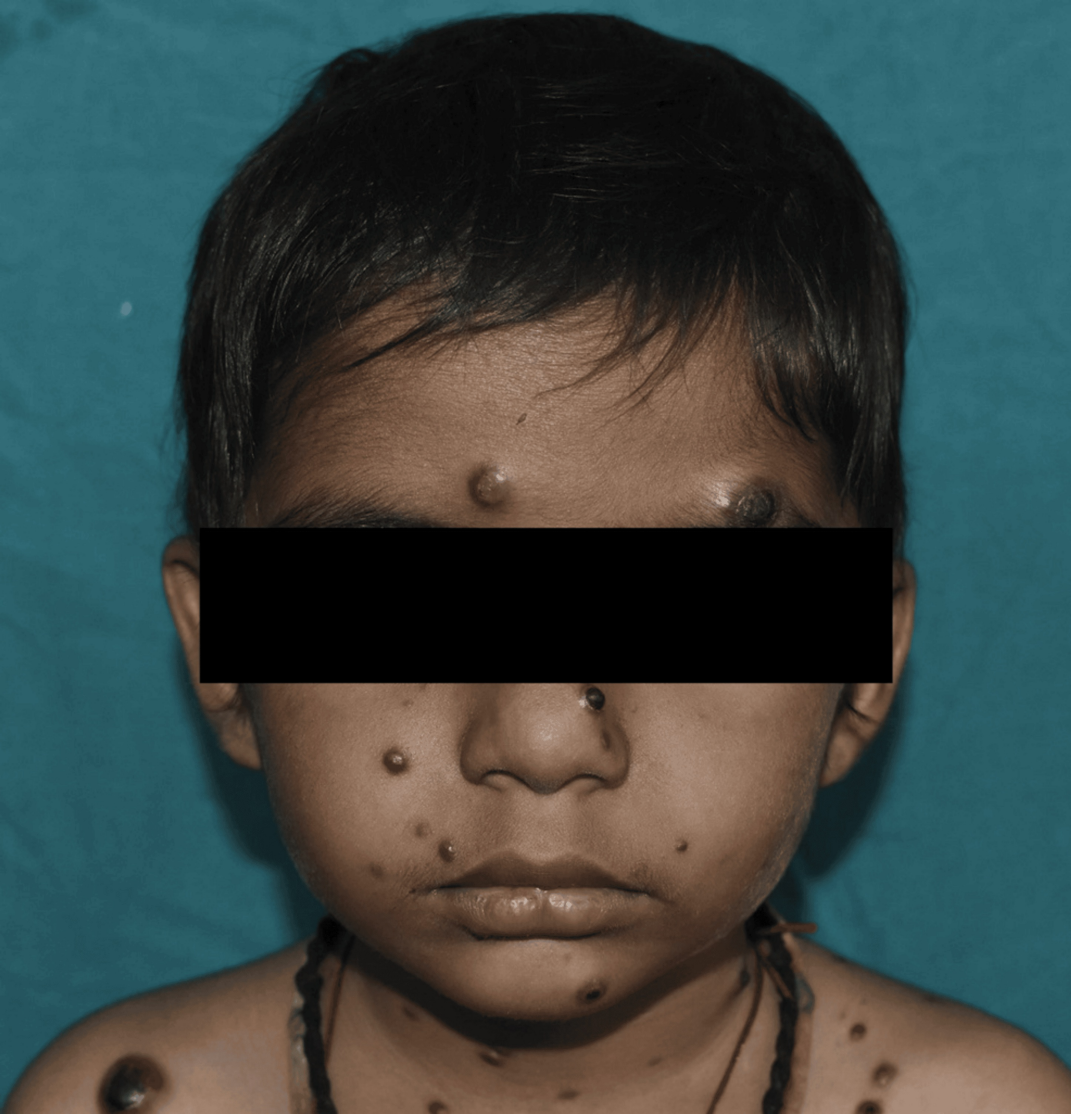
Leukemia cutis: multiple purplish nodules, plaques, and papules over the face and trunk

Similarly, leukemia cutis lesions, which appeared in one case in this study, may indicate an underlying hematological malignancy, with our case involving acute myeloid leukemia, as seen in Figure 8.

## Discussion

In this study of 500 pediatric patients in Gujarat's Saurashtra region, infections and infestations were the most common dermatoses (37%), followed by eczematous disorders (13.4%) and papulosquamous disorders (9.6%). The majority of individuals afflicted were school-aged children (38.4%), with a male-to-female ratio of 1.12:1, indicating a slight male preponderance.

Our study's male preponderance (52.8%) matches the findings of Jawade et al., who found comparable male-to-female ratios in Gujarat, attributing this pattern to boys' increased exposure to outdoor activities [9]. Dogra and Kumar's findings from Northern India support this trend, pointing to environmental and cultural causes [10].

Infections and infestations represented 37% of cases, with bacterial infections accounting for 32.4%. Our results are consistent with those of Reddy et al., who found a 33.8% frequency of infections and infestations in South India [11]. Impetigo has emerged as the most common bacterial infection, according to most studies [12-17]. However, this is in contrast to the findings of Chitapur et al. in South India, where fungal infections were the most prevalent infectious condition, and Hassan et al. in North India, where viral infections were the most prevalent infectious entity [18,19]. These indicate that cold temperatures encourage viral infections, whereas higher temperatures in the south favor fungal infections. Leprosy was observed in 3.4% of cases, reflecting the growing Saurashtra region of Gujarat, which attracts immigrant labor. The affected children in our study were immigrants from Uttar Pradesh and Bihar, both of which are known to be endemic regions for leprosy in India.

Bacterial infections (60; 32.4%) were the most prevalent among infective dermatoses, followed by fungal (52; 28.1%) and viral infections (45; 24.3%), consistent with Balai et al. [20].

Fungal infections accounted for 28.1% of the cases, with tinea corporis and tinea cruris accounting for 50%. Similar findings were reported by Reddy et al., Chitapur et al., and Gupta et al. [11,18,21]. Molluscum contagiosum (26.6%) was the most prevalent viral infection, which was consistent with the findings of Balai et al. [20].

Scabies was the most common parasite infestation (92.8% of cases), which is consistent with Sardana et al. [22].

Eczematous diseases were identified in 13.4% of patients, consistent with Jawade et al. (15.61%) [9]. Our study found that pityriasis alba accounted for 31.3% of eczematous diseases, which is consistent with Hassan et al. [19].

Papulosquamous disorders constituted 9.6% of cases, with pityriasis rosea (31.2%) emerging as the commonest entity. Our findings are consistent with those of Ben Saif and Al Shehab, who discovered that pityriasis rosea was a frequently occurring papulosquamous disorder among children from arid terrain [23]. Our research reported psoriasis incidence (2.4%) corresponds with the findings of Karthikeyan et al., underlining its rarity in Indian pediatric populations [24]. The incidence of lichen planus was discovered to be 2.2%, which is consistent with the findings in Jawade et al. (2.58%), Samman (2%), and Handa and Sahoo (2%) [9,25,26].

Keratinization diseases accounted for 6.2% of cases. This is consistent with Thappa's findings (5.2%) [27]. Palmoplantar keratoderma is the major contributor to this entity followed by lichen spinulosus and +. Nevi and associated disorders accounted for 5.6%, which was close to the 4.55% reported by Gujarati et al. [28].

The higher occurrence of vitiligo (6.4%), keratinization disorders (6.2%), and genodermatoses (2%) in our population can be explained by the fact that our institute is a referral center. Drug eruptions accounted for 1.6%; among them, there were five cases of FDE, TEN due to tenofovir, AGEP due to an unknown drug, and maculopapular rash due to amoxicillin. Sacchidanand et al. reported adverse drug reactions in 0.72% of patients [29].

Our research has a few drawbacks. The study was done in a single center with a limited sample size. To learn more about pediatric dermatoses, a large, prospective multicentric study is required.

The increasing trends of infections and infestations in our study emphasize the need for us to revisit the basic elements of our society such as parents' and children's lack of health awareness, overcrowded living conditions, malnutrition, and inadequate hygiene practices. Addressing these concerns via focused health education campaigns for children and their parents, along with improvements in sanitation, nutrition, and personal hygiene, has the potential to drastically lower the burden of these conditions. Furthermore, frequent school-based health camps may play an important role in the early detection and treatment of skin conditions, reducing the spread of communicable dermatological conditions and enhancing general community health.

## Conclusions

A deep understanding of the patterns of pediatric dermatoses across different geographic regions is essential for promoting improvements in health education, disease control, and preventive initiatives tailored to specific communities. This knowledge not only facilitates meaningful comparisons with data from other regions in India and globally but also highlights the pressing need for action. The significant prevalence of infectious dermatoses identified in our study calls for immediate and focused hygiene education, along with appropriate clinical care. Additionally, the seasonal and geographic variations in dermatoses strongly indicate that we must adopt climate-specific preventive measures and robust public health strategies to effectively address these challenges.
